# Supplementary material for: AmiR-P3: An AI-based microRNA prediction pipeline in plants
Source: PLoS One. 2024 Aug 1;19(8):e0308016. doi: 10.1371/journal.pone.0308016 (PMC11293646; doi:10.1371/journal.pone.0308016)
Supplement: S1 Table — comprehensive information about some of the available plant miRNA prediction tools, including the prediction methods and necessary inputs. (DOCX) [file pone.0308016.s001.docx]

**AmiR-P^3^: An AI-based microRNA prediction pipeline in plants**

Sobhan Ataei, Jafar Ahmadi, Sayed-Amir Marashi, Ilia Abolhasani

**S1 Table**

**An overview of the available tools for predicting plant miRNAs**

| **Software name** | **method** | **Necessary inputs** | **Tested on** | **Reference** | **Availability** |
| --- | --- | --- | --- | --- | --- |
| miRNAFinder1 [1] | multilayer perceptron (MLP) based classifier | - NGS reads - Reference genome - ncRNA sequences | *B. distachyon, G. max, G. raimondii, L. japonicus, M. truncatula, O. sativa, P. abies, P. trichocarpa, S. bicolor, and S. tuberosum* | Lokuge, Jayasundara et al. (2022) | Online/Local |
| PlantMirP2 [2] | SVM | - Simple FASTA sequence for pre-microRNA prediction. - NGS data file, genome data file, and ncRNA data file for mature microRNA prediction | 5323 pre-miRNAs belonging to all of the plant entries in the miRBase (release 22.1) except for *A. thaliana, G. max, O. sativa, P. patens, M. Truncatula, S. bicolor, A. lyrata, Z. mays,* and *S. lycopersicum* | Fan, Yao et al. (2021) | Online/Local |
| mirMachine [3] | Rule-based | - Formatted NGS reads - Reference genome | *A. thaliana*  *T. aestivum* | Cagirici, Sen et al. (2021) | Online/Local |
| PmiRDiscVali2 [4] | Based on miRDeep-P | - sRNA NGS reads - RNA NGS reads - Optional degradome NGS reads - Optional reference genome | *D. officinale* | Yu, Wan et al. (2019) | Local |
| miRDeep-P2 (miRDP2) [5] | Random Forest (RF) based classifier | - NGS formatted reads - Reference genome | *A. thaliana, O. sativa, S. lycopersium, Z. mays, T. aestivum* | Kuang, Wang et al. (2019) | Local |
| SUmir2 [6] | Rule-based | - High-throughput genomic and transcriptomic sequences in FASTA format or sRNA sequencing data | *B. distachyon*  *T. aestivum* | Alptekin, Akpinar et al. (2017) | Local |
| miRNA Digger3 [7] | Rule-based | - Degradome sequencing data - Reference genome | *Arabidopsis* | Yu, Shao et al. (2016) | Local |
| miRPlant4 [8] | Based on miRDeep* | - NGS sRNA reads - Reference genome | *A. thaliana*  *M. truncatula*  *P. persica* | An, Lai et al. (2014) | Local |
| miPlantPreMat [9] | SVM | - Simple FASTA sequence | *A. thaliana, G. max, O. sativa, P. patens, M. truncatula, S. bicolor, A. lyrata, Z. mays, and S. lycopersicum* | Meng, Liu et al. (2014) | Local |
| miR-PREFeR [10] | Rule-based | - NGS sRNA reads - Reference genome - Optional annotation file | *A. thaliana* | Lei and Sun (2014) | Local |
| C-mii [11] | Rule-based | - Simple FASTA sequence | *A. thaliana* | Numnark, Mhuantong et al. (2012) | Local |

1. This tool employs a multilayer perceptron (MLP) based classifier implemented using 180 features under sequential, structural, and thermodynamic feature categories for plant pre-miRNA identification. This classifier is reported to have 92% accuracy, 94% specificity, and 90% sensitivity.
2. This tool Uses miRDeep-P [12] as the core prediction algorithm.
3. miRNA Digger is developed for genome-wide extraction of miRNA candidates by searching for the degradome-supported miRNA processing sites. This program cannot be applied to the species without a reference genome.
4. miRPlant is a derivate of miRDeep* [13] which is specifically adjusted and tuned for predicting plant miRNAs . The use of miRPlant is highly dependent on the availability of genomic data, indicating the inappropriateness of this tool for miRNA prediction based on RNA-seq data.

**References:**

1. Lokuge S, Jayasundara S, Ihalagedara P, Kahanda I, Herath D. miRNAFinder: A comprehensive web resource for plant Pre-microRNA classification. Biosystems. 2022;215-216:104662.

2. Fan D, Yao Y, Yi M. PlantMirP2: An Accurate, Fast and Easy-To-Use Program for Plant Pre-miRNA and miRNA Prediction. Genes. 2021;12(8):1280.

3. Cagirici HB, Sen TZ, Budak H. mirMachine: a one-stop shop for plant miRNA annotation. JoVE (Journal of Visualized Experiments). 2021(171):e62430.

4. Yu D, Wan Y, Ito H, Ma X, Xie T, Wang T, et al. PmiRDiscVali: an integrated pipeline for plant microRNA discovery and validation. BMC genomics. 2019;20(1):133.

5. Kuang Z, Wang Y, Li L, Yang X. miRDeep-P2: accurate and fast analysis of the microRNA transcriptome in plants. Bioinformatics (Oxford, England). 2019;35(14):2521-2.

6. Alptekin B, Akpinar BA, Budak H. A Comprehensive Prescription for Plant miRNA Identification. Frontiers in plant science. 2017;7(2058):2058.

7. Yu L, Shao C, Ye X, Meng Y, Zhou Y, Chen M. miRNA Digger: a comprehensive pipeline for genome-wide novel miRNA mining. Scientific reports. 2016;6(1):18901.

8. An J, Lai J, Sajjanhar A, Lehman ML, Nelson CC. miRPlant: an integrated tool for identification of plant miRNA from RNA sequencing data. BMC bioinformatics. 2014;15(1):275.

9. Meng J, Liu D, Sun C, Luan Y. Prediction of plant pre-microRNAs and their microRNAs in genome-scale sequences using structure-sequence features and support vector machine. BMC bioinformatics. 2014;15(1):423.

10. Lei J, Sun Y. miR-PREFeR: an accurate, fast and easy-to-use plant miRNA prediction tool using small RNA-Seq data. Bioinformatics (Oxford, England). 2014;30(19):2837-9.

11. Numnark S, Mhuantong W, Ingsriswang S, Wichadakul D. C-mii: a tool for plant miRNA and target identification. BMC genomics. 2012;13(7):S16.

12. Yang X, Li L. miRDeep-P: a computational tool for analyzing the microRNA transcriptome in plants. Bioinformatics (Oxford, England). 2011;27(18):2614-5.

13. An J, Lai J, Lehman ML, Nelson CC. miRDeep*: an integrated application tool for miRNA identification from RNA sequencing data. Nucleic Acids Research. 2013;41(2):727-37.
