# Supplementary material for: AmiR-P3: An AI-based microRNA prediction pipeline in plants
Source: PLoS One. 2024 Aug 1;19(8):e0308016. doi: 10.1371/journal.pone.0308016 (PMC11293646; doi:10.1371/journal.pone.0308016)
Supplement: S2 File — The software tool employed for extracting features from the predicted secondary structures of confirmed miRNAs, decoy sequences and candidate pri-miRNAs. (DOCX) [file pone.0308016.s005.docx]

**AmiR-P^3^: An AI-based microRNA prediction pipeline in plants**

Sobhan Ataei, Jafar Ahmadi, Sayed-Amir Marashi, Ilia Abolhasani

**S2 File**

**An introduction to CTAnalyzer**

CTAnalyzer is a simple software tool developed in Python programming language for extracting features from the secondary structures of confirmed and candidate pri-miRNAs. The extracted features from experimentally-validated pri-miRNAs can then be used to train miRNA prediction algorithms or as selection criteria in rule-based utilities. Subsequently, by extracting the features of the secondary structure of each candidate miRNA and utilizing them in the previously mentioned tools, it will be possible to decide whether the predicted structure can be considered as a pri-miRNA [1].

Our approach for *ab initio* prediction of plant miRNAs is based on features extracted from the predicted secondary structures of candidate RNA sequences. Various RNA secondary structure prediction tools (*e.g.,* mFold [2] and RNA structure [3]), store their output in the .CT (connectivity table) file format. Each .CT file summarizes the secondary structure information of an RNA molecule. For a predicted RNA secondary structure, the corresponding .CT file follows the following format (Fig. S2.1): The first line starts with the length and ends with the given title of the sequence. The following lines contain information about each residue in the sequence. For the *n*’th residue of the RNA molecule (in 5’ to 3’ order), line *n*+1 in the .CT file represents the following data in order:

1. Nucleotide number: index *n*
2. Nucleotide type (A, T, G, C, U)
3. Index *n*-1
4. Index *n*+1
5. Number of the nucleotide to which the *n*’th nucleotide is paired. A zero value means that the *n*’th nucleotide is not paired to any other nucleotide in the RNA secondary structure.
6. Natural nucleotide numbering in the original sequence


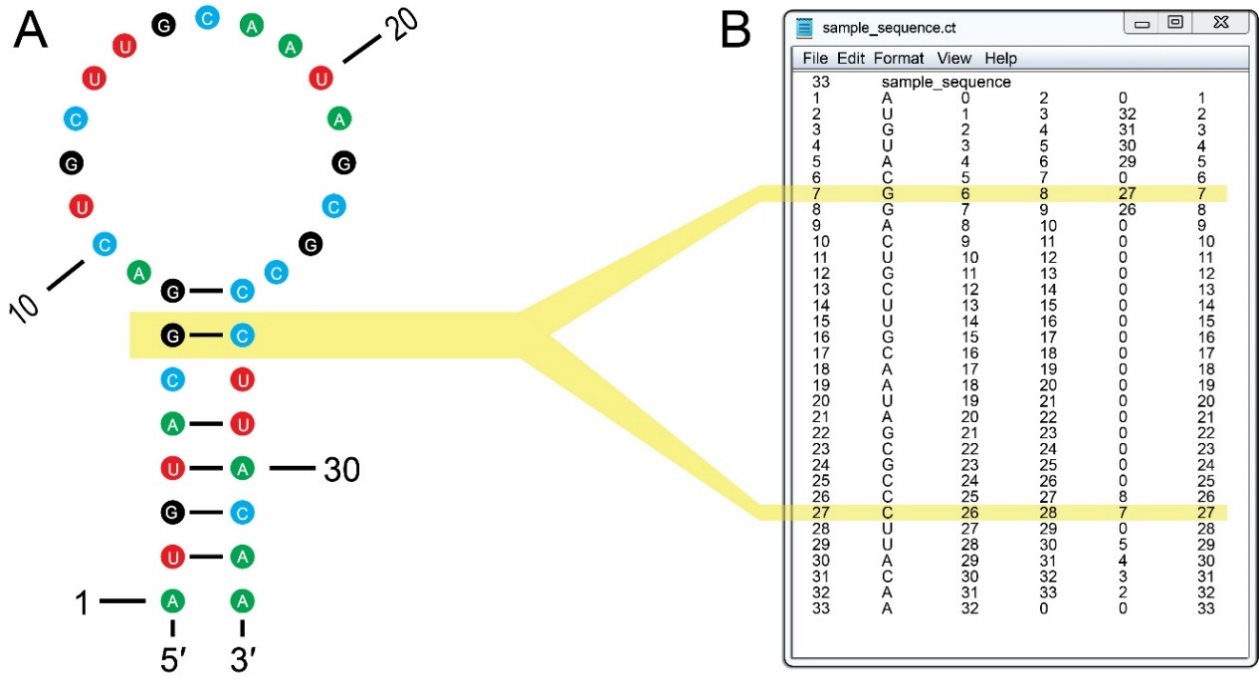


**Fig. S2.1.** Visualization of a predicted RNA secondary structure and its corresponding .CT file. As shown in (A), nucleotides in the 2^nd^, 3^ed^, 4^th^, 5^th^, 7^th^ and 8^th^ positions are paired to 32^nd^, 31^st^, 30^th^, 29^th^, 27^th^ and 26^th^ nucleotides respectively. These structural data are also available from the .CT file (B). In the .CT file, the first line starts with the length of the RNA molecule and ends with its given ID. The rest of the file consists of six columns. Line (n+1) in the file refers to the n’th nucleotide and includes the nucleotide number (index n), nucleotide type (A, T, G, C, U), Index n-1, Index n+1, number of the nucleotide to which the *n’*th nucleotide is paired, and the natural numbering in the original sequence. As an illustrative example, base-pairing of the 7^th^ and the 27^th^ nucleotides are highlighted here.

As the name suggests, CTAnalyzer accepts .CT files as its input. Suppose we have a pri-miRNA candidate sequence, and we choose a certain segment of this sequence (the “hit region” in the present work) as the miRNA sequence. Then, by extracting features from the predicted secondary structure of this RNA, CTAnalyzer verifies whether the structure resembles a pri-miRNA, that is, whether it has the characteristics of known plant miRNAs. More precisely, if a candidate miRNA and its complementary segment (*i.e.,* miRNA*) are located on a double-stranded RNA hairpin structure with near-perfect complementarity and without inner branches (neither on the miRNA nor the miRNA*), then this candidate sequence has the structural characteristics of a putative pri-miRNA. In contrast, the predicted structure is not accepted if the hit region has any of these characteristics:

(1) It is not involved in a double-stranded stem (Fig. S2.2A); or

(2) It has only a few residues in complementarity (Fig. S2.2B); or

(3) It lacks a continuous complementary region (Fig. S2.2C); or

(4) It contains inner branches (Fig. S2.2D); or

(5) It is complementary to a branched region (Fig. S2.2E); or

(6) It is not located entirely in the same side of the duplex (Fig. S2.2F).


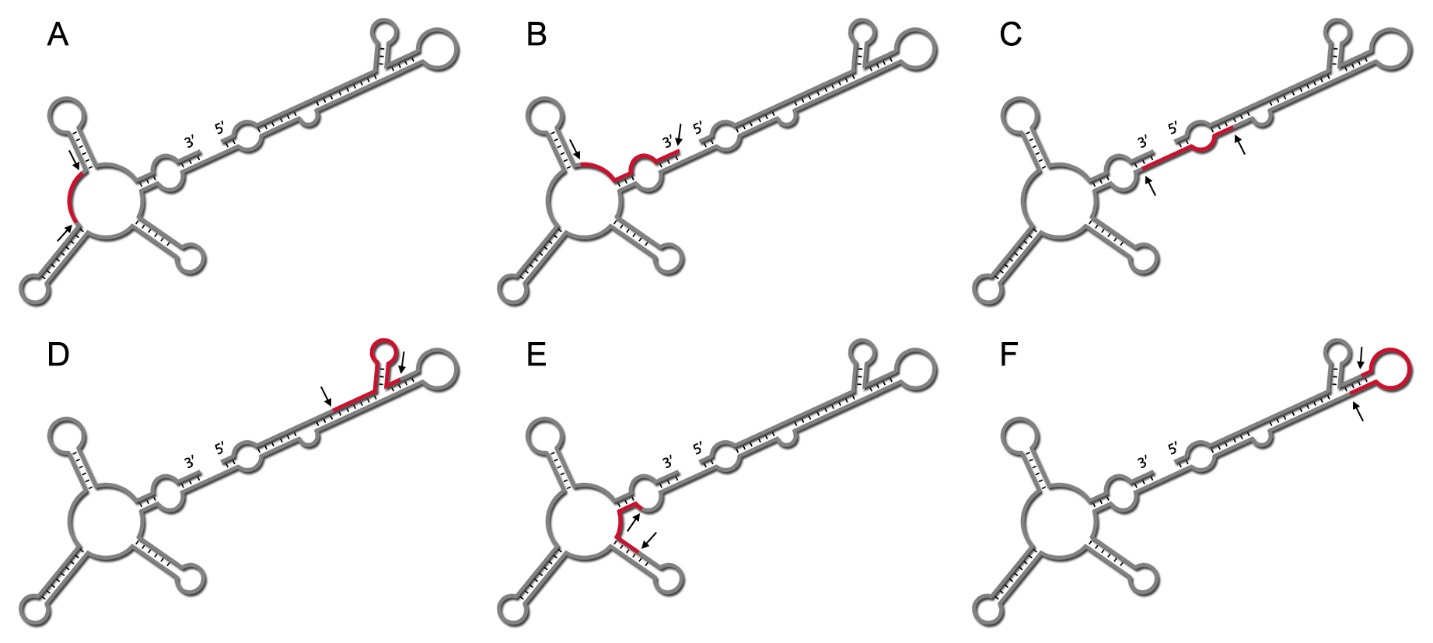


**Fig. S2.2.** Illustrative examples of predicted secondary structures that are not acceptable as pri-miRNAs. In each panel, the overall structure is shown in gray, while the colored segment is assumed to represent the hit region. These structures cannot be considered acceptable pri-miRNAs because: (A) the hit region is not part of a double-stranded segment; (B) the hit region lacks a near-perfect complementarity; (C) the complementary of the hit region is discontinuous; (D) the hit region has an inner branch; (E) the complementary of the hit sequence has several inner structures; (F) the hit region is not located in just one arm of the duplex structure.

In contrast to their animal counterparts, plant pri-miRNAs are known to be much longer and possess up to 300 nucleotides [4]. Since in homology-based miRNA prediction approaches, the exact position of the hit region on the candidate pre-miRNA is not known, a relatively wide genomic window must be chosen around the hit region to guarantee the inclusion of the complete pri-miRNA sequence. Consequently, it is not surprising to obtain a predicted RNA secondary structure that has little similarity to a classical pri-miRNA stem-loop structure. As demonstrated in Fig S2.3, in the predicted multi-branched structure, CTAnalyzer recognizes the main branch, which includes the miRNA/miRNA* duplex (which will be referred to as the “branch of interest”, or BOI for short), and extracts it from the rest of the structure for further evaluation and then, for the feature extraction process.

As demonstrated in Fig S2.3, CTAnalyzer partitions the extracted branch into three main segments, *i.e.,* the loop-proximal region, the hit region, and the loop-distal region.


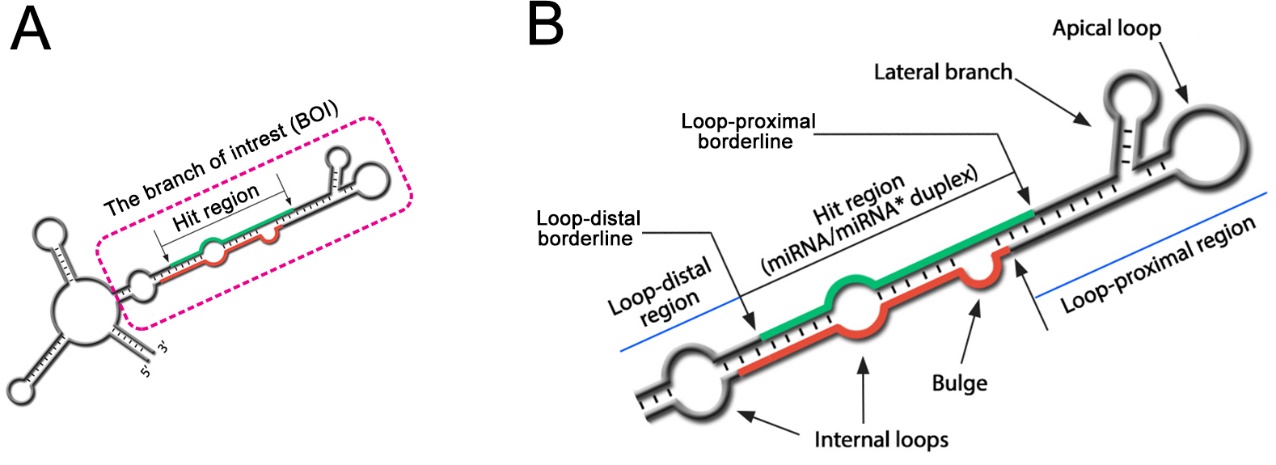


**Fig. S2.3.** An illustrative example of a predicted secondary structure of a pri-miRNA and the extracted BOI from it. (A) CTAnalyzer verifies and extracts the main branch (BOI), which includes the putative miRNA/miRNA* duplex. This branch will then be used in the feature extraction process. (B) Three main segments of the BOI, i.e., the loop-proximal region, the hit region, and the loop-distal region, are demonstrated in the BOI. A bulge, two internal loops, a lateral branch, and an apical loop are also shown.

While analyzing the BOI, CTAnalyzer finds answers to the three main questions about structural features: (*i*) What features are present in the BOI? (*ii*) From which part of the structure are these features extracted? (*iii*) What are the properties of the identified features?

**Types** **of structural features:** CTAnalyzer considers four main types of structural features, namely mismatches, bulges, internal loops, and lateral branches. A bulge is a structural motif formed in a double-stranded part of the structure in which at least one nucleotide of one strand is unpaired. Internal loops are motifs characterized by unpaired nucleotides in both strands. The motif is known as a symmetric internal loop if the number of unpaired nucleotides is equal for both strands. Otherwise, an asymmetric internal loop is formed [5, 6]. By default, motifs containing more than two consecutive mismatches are categorized as symmetric internal loops by CTAnalyzer, although this threshold value is adjustable by the user. Lateral branches (as illustrated in Fig S2.3B) are rare features observed, for example, in miR319/159 [4]. In most miRNA prediction approaches, structures with lateral branches are discarded. By default, the maximum acceptable number for lateral branches in the loop-proximal region is set to 1 in CTAnalyzer, but this threshold is also adjustable by the user (from zero to greater values).

**Location of structural features:** After BOI recognition, considering the location of the miRNA/miRNA* duplex and the terminal structure (apical loop), three sections of the BOI are inspected: the hit region (or, more precisely, the miRNA/miRNA* duplex), the loop-proximal region, and the loop-distal region, as shown in Fig S2.3B. Consequently, all the extracted structural and sequence features are linked to one of the regions mentioned above. Since two adjacent regions might be associated with the same structural feature (for example, consecutive mismatches, an asymmetric bulge, or an internal loop), two borderline regions (*i.e.,* loop-proximal borderline and loop-distal borderline), which are the DCL cut sites in a pri-miRNA, are also considered in the feature extraction process.

**Properties of the identified features:** After locating the above-mentioned features, their properties are investigated. For instance, the number of nucleotides in consecutive mismatches (which form symmetric internal loops), the number of bulged residues and the number of unpaired residues on each strand of an internal loop, the length of the loop-distal and loop-proximal stems, the number of residues involved in the apical loop, and, the number and the length of the lateral branches are determined.

**Structural hot****spots:** Some parts of pri-miRNAs are known (or at least suggested) to play essential roles in miRNA biogenesis. These important segments of the RNA secondary structure are termed “structural hotspots” in CTAnalyzer. For instance, it is believed that the first DCL cleavage occurs approximately 15 base pairs away from a large internal loop in the loop-distal or the loop-proximal regions, toward the miRNA/miRNA* duplex, depending on the direction of miRNA processing (*i.e.,* base to loop or loop to base) [7, 8]. Consequently, the 15 bp regions in the loop-proximal and the loop-distal regions are considered as two structural hotspots on the secondary structure of the pri-miRNA. As a further example, due to its essential role in the functionality of the miRNA, the seed region on the mature miRNA (*i.e.,* the 2^nd^ toward the 13^th^ nucleotide of the hit region [9]) is considered as another hotspot and should be inspected for specific sequence and/or structure patterns.

**Nucleotide distribution and mismatch compositions:** Primary miRNAs are not reported to possess conserved sequences outside the mature miRNA region [10]. However, some studies have shown that even in these regions, certain segments may have distinct nucleotide distributions (*e.g*., in a few residues before and after the first Dicer cut site [11]). It is also suggested that some mismatch compositions (*e.g.,* C-C mismatches) are less frequent in specific regions [12], and one can conclude that mismatch compositions may include important biological information. Consequently, CTAnalyzer also extracts the nucleotide distribution and mismatch compositions of the crucial segment of the pri-miRNA, *i.e*., the structural hotspots.

**Energy features:** Since pri-miRNAs are known to form stable molecular structures, it is important to include the calculated free energy of the structure as a feature in the miRNA prediction process. Using the minimum free energy (MFE) of the predicted structure, CTAnalyzer calculates the adjusted minimum free energy (AMFE) and the minimum free energy index (MFEI) of the predicted secondary structure.

Following is a summary of extracted features for each .CT file:

**Major structural features of the predicted structures:** including Lateral structures, mismatches, bulges, and symmetric and asymmetric internal loops in five sections of the predicted structure (the hit region, loop-proximal and loop-distal regions, loop proximal and loop-distal regions) and the distance of effective and interfering internal structures from Structural hot spots and critical regions.

**Sequence features:** including the sequence and relative position of the hit region and its complementary sequence on the predicted structures, and the location and the sequence of the predicted precursor.

**Nucleotide frequency and mismatch arrangement:** including the GC content of the predicted structure, BOI, and the predicted precursor. Composition of matched and mismatched residues in Structural hot spots and critical regions.

**Energy features:** including MFE, AMFE, and MFEI, calculated for the complete predicted structure, BOI, and the predicted pre-miRNA.

**References**

1. Ataei S, Ahmadi J, Marashi S-A. Utilization of CTAnalyzer algorithm to identify pri-microRNA candidate sequences in the genome of *Azadirachta indica*. Modern Genetics Journal. 2021;16(4):349-61. (in Persian).

2. Zuker M. Mfold web server for nucleic acid folding and hybridization prediction. Nucleic Acids Research. 2003;31(13):3406-15.

3. Reuter JS, Mathews DH. RNAstructure: software for RNA secondary structure prediction and analysis. BMC bioinformatics. 2010;11:129.

4. Axtell MJ, Meyers BC. Revisiting Criteria for Plant MicroRNA Annotation in the Era of Big Data. The Plant Cell. 2018;30(2):272-84.

5. Tian B, Bevilacqua PC, Diegelman-Parente A, Mathews MB. The double-stranded-RNA-binding motif: interference and much more. Nature Reviews Molecular Cell Biology. 2004;5(12):1013-23.

6. Popenda M, Szachniuk M, Blazewicz M, Wasik S, Burke EK, Blazewicz J, et al. RNA FRABASE 2.0: an advanced web-accessible database with the capacity to search the three-dimensional fragments within RNA structures. BMC bioinformatics. 2010;11(1):231.

7. Bologna NG, Schapire AL, Palatnik JF. Processing of plant microRNA precursors. Brifings in functional genomics. 2012;12(1):37-45.

8. Zhu H, Zhou Y, Castillo-González C, Lu A, Ge C, Zhao Y-T, et al. Bidirectional processing of pri-miRNAs with branched terminal loops by Arabidopsis Dicer-like1. Nature structural and molecular biology. 2013;20(9):1106-15.

9. Dai X, Zhuang Z, Zhao PX. psRNATarget: a plant small RNA target analysis server (2017 release). Nucleic Acids Research. 2018;46(W1):W49-W54.

10. Reinhart BJ, Weinstein EG, Rhoades MW, Bartel B, Bartel DPJG, development. MicroRNAs in plants. GENES & DEVELOPMENT. 2002;16(13):1616-26.

11. Miskiewicz J, Tomczyk K, Mickiewicz A, Sarzynska J, Szachniuk M. Bioinformatics Study of Structural Patterns in Plant MicroRNA Precursors. BioMed research international. 2017;2017:6783010.

12. Rojas AML, Drusin SI, Chorostecki U, Mateos JL, Moro B, Bologna NG, et al. Identification of key sequence features required for microRNA biogenesis in plants. Nature Communications. 2020;11(1):5320.
