# Supplementary material for: AmiR-P3: An AI-based microRNA prediction pipeline in plants
Source: PLoS One. 2024 Aug 1;19(8):e0308016. doi: 10.1371/journal.pone.0308016 (PMC11293646; doi:10.1371/journal.pone.0308016)
Supplement: S3 File — Sequence logo illustrations to examine the potential bias resulting from the occurrence of nucleotide patterns in the flanking sequences of genuine and decoy pre-miRNAs within the positive and negative datasets. (DOCX) [file pone.0308016.s006.docx]

**AmiR-P^3^: An AI-based microRNA prediction pipeline in plants**

Sobhan Ataei, Jafar Ahmadi, Sayed-Amir Marashi, Ilia Abolhasani

**S3 File**

**Sequence Logos of 20nt Flanking Regions in Positive and Negative pre-miRNA Datasets**

To evaluate the potential bias caused by the nucleotide patterns in the neighboring sequences of genuine and simulated pre-miRNAs in the positive and negative datasets, we used WebLogo (version 3.7.12) to generate sequence logos (Fig. S3.1). This representation is to show the level of similarity among sequences in a certain set, *e. g.*, the set of downstream and upstream flanking sequences in pre-miRNAs. The more the conservedness of the sequences at each position, the greater the information of the nucleotides at that position.

Figures S3.2-S3.5 show the sequence logos of the 20 nucleotides that are downstream and upstream of each real or decoy pre-miRNA datasets. Fig. S3.2 and Fig. S3.3 represent the 20 nucleotides downstream and upstream of pre-miRNAs (in the positive dataset), respectively. Fig. S3.4 and Fig. S3.5 represent the 20 nucleotides downstream and upstream of decoy pre-miRNAs (in the negative dataset), respectively. These logos indicate that there is no significant nucleotide conservation in the flanking regions in either dataset.


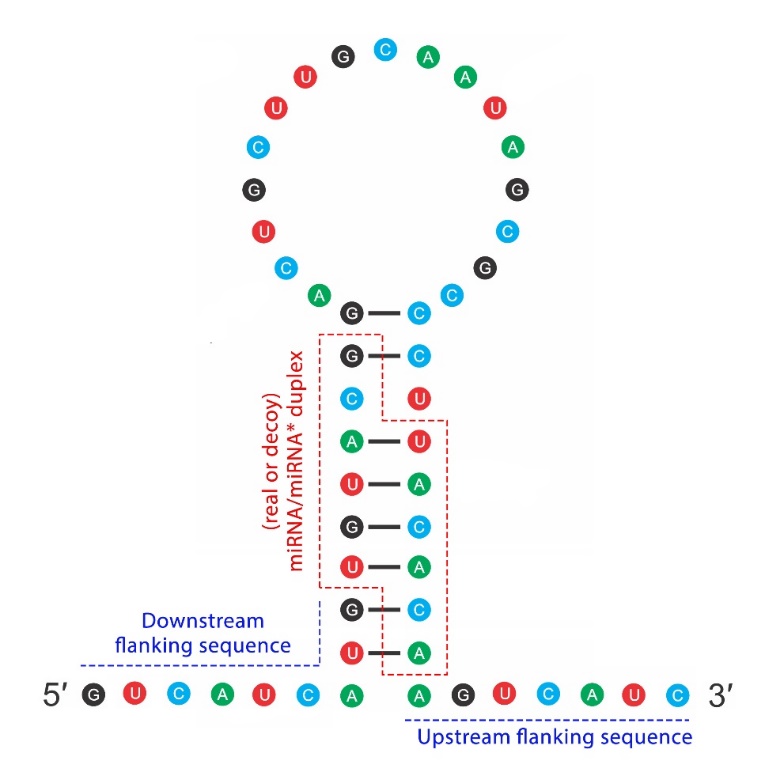


**Fig. S3.1.** Visualization of a real/decoy pre-microRNA with two flanking sequences


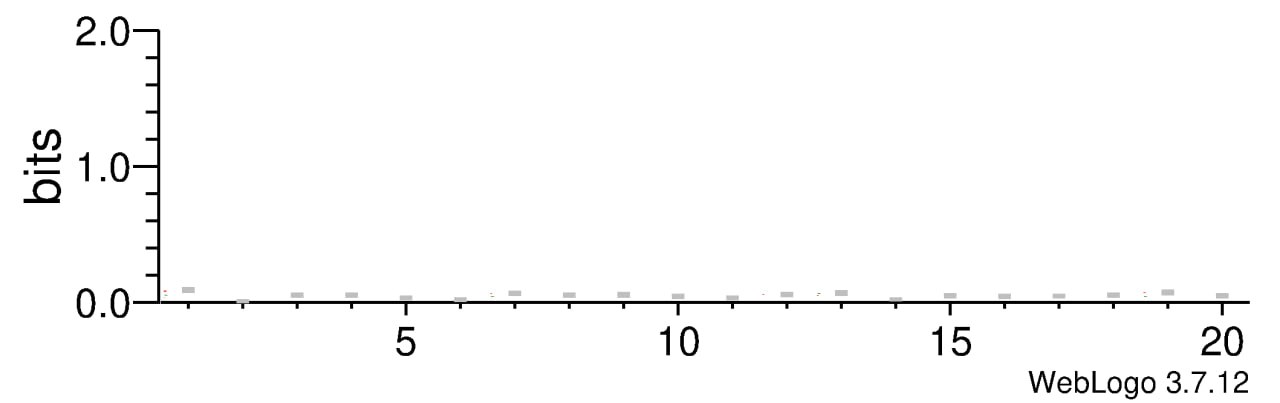


**Fig. S3.2.** WebLogo diagram for the 20 nucleotides downstream of the pre-miRNAs in the positive datasets. The height of each column at each position represents the information level at that position (in bits).


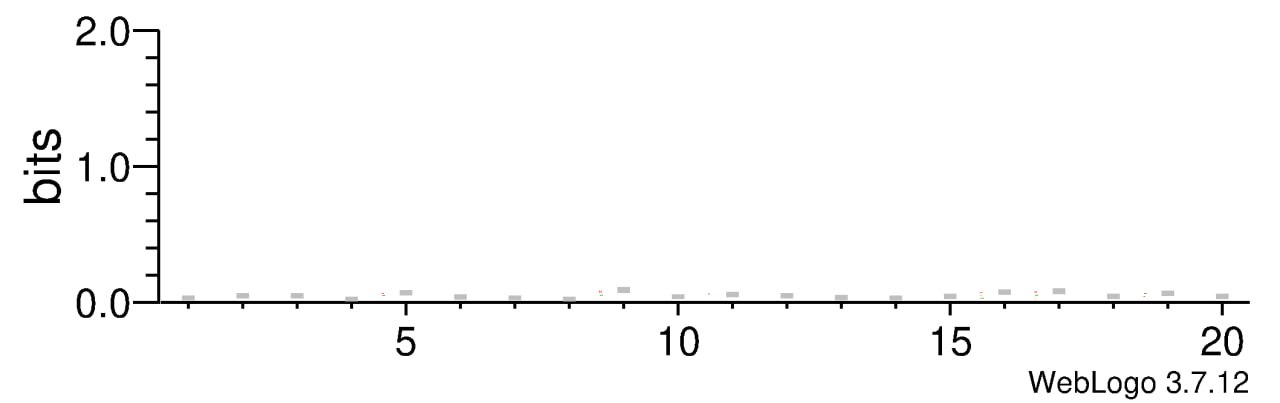


**Fig. S3.3.** WebLogo diagram for the 20 nucleotides upstream of the pre-miRNAs in the positive datasets. The height of each column at each position represents the information level at that position (in bits).


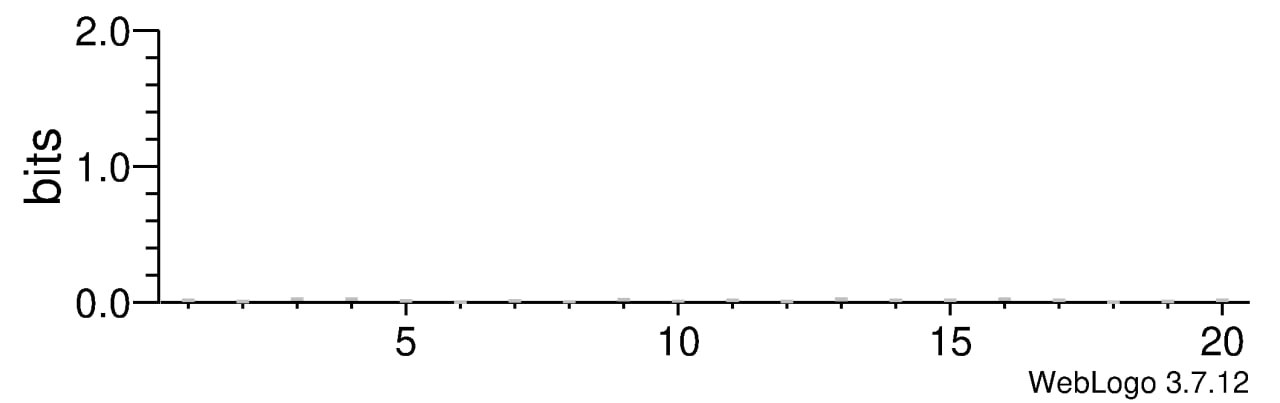


**Fig. S3.4.** WebLogo diagram for the 20 nucleotides downstream of the decoy pre-miRNAs in the negative datasets. The height of each column at each position represents the information level at that position (in bits).


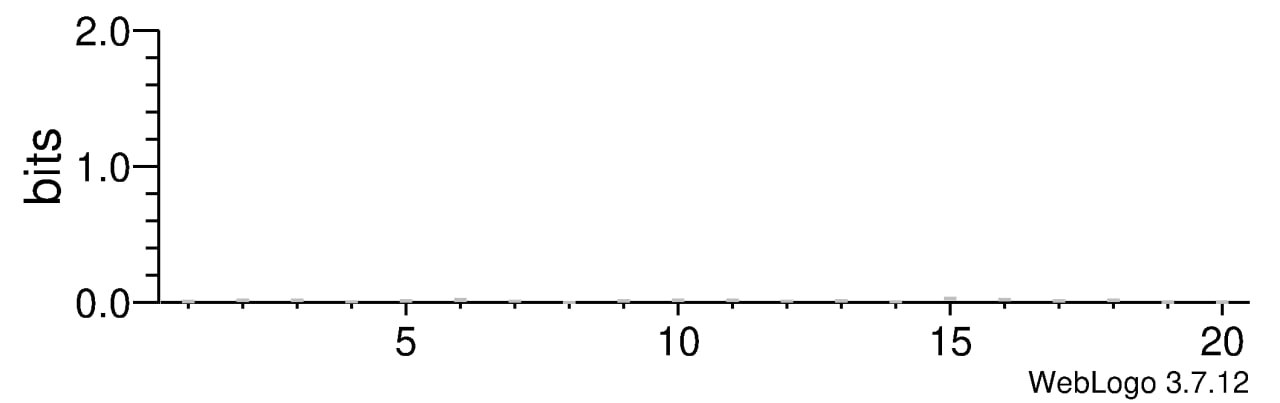


**Fig. S3.5.** WebLogo diagram for the 20 nucleotides upstream of the decoy pre-miRNAs in the negative datasets. The height of each column at each position represents the information level at that position (in bits).

To better understand the results, we also used WebLogo to calculate the level of conservation in each sequence set as the percentage of nucleotides at each position. Figures S3.6-S3.9 show the results of this analysis, which suggest that each set has a more-or-less uniform distribution of nucleotides along the 20 nt sequence. This observation confirms that the percentage of the four nucleotides at each position is mainly determined by the inherent nucleotide composition of the dataset at that region, and no specific conservedness pattern is observed among sequences.


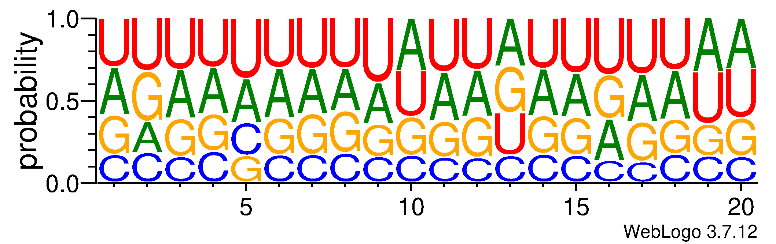


**Fig. S3.6.** WebLogo diagram for the 20 nucleotides downstream of the pre-miRNAs in the positive datasets. The height of each column at each position represents the information level at that position (in percentage of nucleotides).


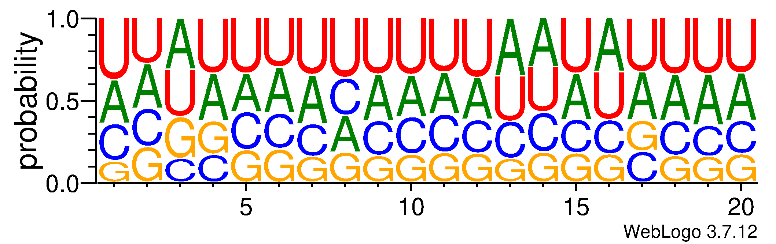


**Fig. S3.7.** WebLogo diagram for the 20 nucleotides upstream of the pre-miRNAs in the positive datasets. The height of each column at each position represents the information level at that position (in percentage of nucleotides).


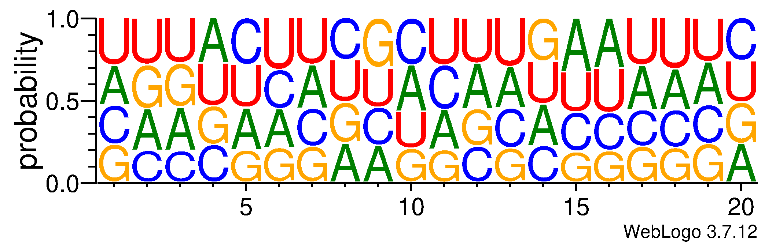


**Fig. S3.8.** WebLogo diagram for the 20 nucleotides downstream of the decoy
pre-miRNAs in the negative datasets. The height of each column at each position represents the information level at that position (in percentage of nucleotides).


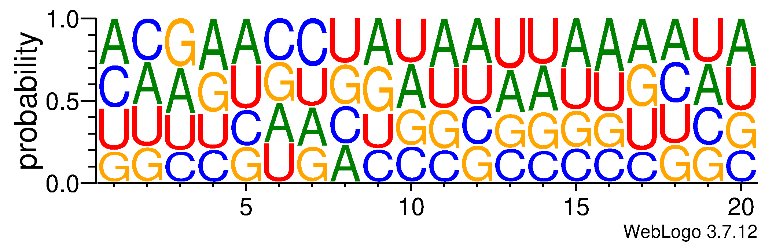


**Fig. S3.9.** WebLogo diagram for the 20 nucleotides upstream of the decoy
pre-miRNAs in the negative datasets. The height of each column at each position represents the information level at that position (in percentage of nucleotides).
