# Supplementary material for: AmiR-P3: An AI-based microRNA prediction pipeline in plants
Source: PLoS One. 2024 Aug 1;19(8):e0308016. doi: 10.1371/journal.pone.0308016 (PMC11293646; doi:10.1371/journal.pone.0308016)
Supplement: S4 File — Details on the developed neural network employed to classify the predicted RNA secondary structures. (DOCX) [file pone.0308016.s007.docx]

**AmiR-P^3^: An AI-based microRNA prediction pipeline in plants**

Sobhan Ataei, Jafar Ahmadi, Sayed-Amir Marashi, Ilia Abolhasani

**S4 File**

**Details on the implemented neural network**

In this study, we developed a neural network for a classification task that utilized seven SELU dense layers with dropout regularization and batch normalization to mitigate overfitting and vanishing gradients while accelerating training (See Fig 1). The final layer of the model was a softmax layer that provided probability scores for each class, enabling accurate classification of RNA sequences as either miRNA or decoys.

We evaluated our model using a 9-fold cross-validation approach, that is, selecting one plant species as the test set while using the other eight species to train the model, with a random 33% split of the training data reserved for validation purposes. As soon as the training is complete, we tested the correctness of the predictions based on th remaining dataset, that is, the nineth plant. This process was repeated for all nine plant species, ensuring complete independence between the test and train datasets and demonstrating the model's ability to generalize across species.

We trained our model using Adam optimization with an exponential decay learning rate, initially set to 0.002 with a decay rate of 0.99, and a batch size of 32. Upon completing the cross-validation process, we trained the model on all miRNA datasets which correspond to all of the nine species. Then, the model was integrated into our pipeline for classifying the predicted RNA structures.

Our results demonstrated the high effectiveness of our deep learning model in differentiating genuine miRNA from decoy. The model's ability to generalize across species is a significant strength, as it allows for the identification of miRNA targets across multiple plant species. Overall, our approach provides a valuable tool for identifying miRNA sequences and predicting potential miRNA targets in various plant species including the orphan, less-studied plants. A schematic representation of the neural network model to classify miRNA and decoy sequences is presented in Fig. S4.1.


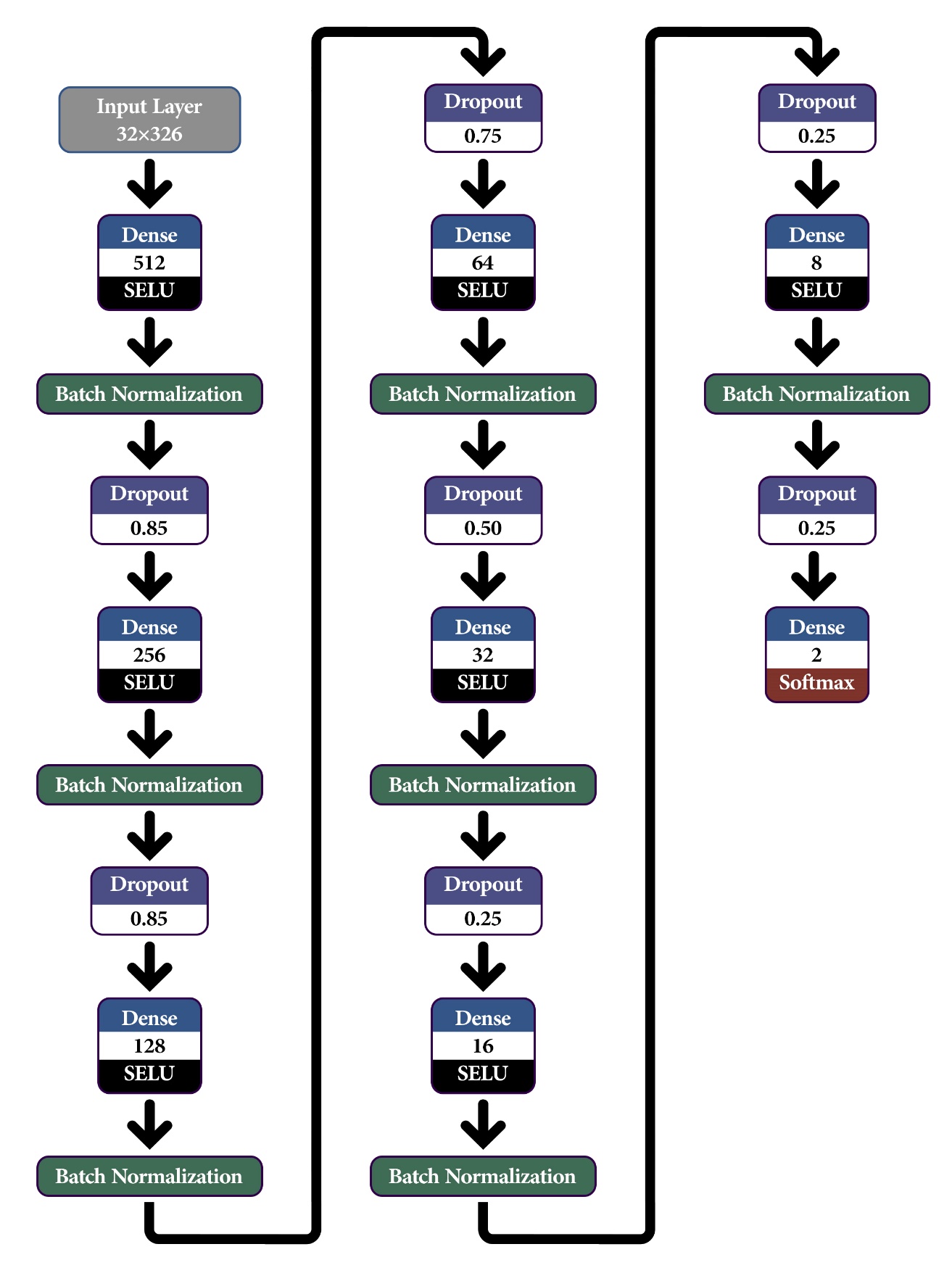


**Fig. S4.1.** Schematic representation of the neural network model to classify miRNA and decoy sequences.
