# Supplementary material for: AmiR-P3: An AI-based microRNA prediction pipeline in plants
Source: PLoS One. 2024 Aug 1;19(8):e0308016. doi: 10.1371/journal.pone.0308016 (PMC11293646; doi:10.1371/journal.pone.0308016)
Supplement: S5 File — Details on general structure and prediction approach of AmiR-P3. (DOCX) [file pone.0308016.s008.docx]

**AmiR-P^3^: An AI-based microRNA prediction pipeline in plants**

Sobhan Ataei, Jafar Ahmadi, Sayed-Amir Marashi, Ilia Abolhasani

**S5 File**

**Technical details of AmiR-P^3^ pipeline**

AmiR-P^3^ is designed to predict candidate miRNAs in (non-)annotated genomic or transcriptomic sequence(s). The set of genomic or transcriptomic sequences should be provided as an input file in FASTA (or, multi-FASTA) format. Apart from this file, to run AmiR-P^3^, two datasets are required: the set of known plant miRNAs, and the set of all known protein sequences. In the current version, by default, all mature miRNA sequences of Viridiplantae from miRBase (Release 22.1) are included in the pipeline (and the new updates are automatically downloaded upon installation). The user can select a subset of these sequences based on species and/or level of annotation confidence. After this step, identical miRNA sequences are grouped into clusters by cd-hit-est. Then, each sequence of this dataset is considered as a query for the initial homology search. The second essential dataset is a comprehensive protein dataset (*e.g.,* NCBI-NR, that is, the non-redundant set of proteins in NCBI GenBank). A comprehensive set of all known proteins is required for sequence alignment, to ensure that the found miRNA-resembling sequences do not overlap protein–coding regions.

**Data input and homology search**

As mentioned before, AmiR-P^3^ accepts genomic or transcriptomic sequences in a (multi-)FASTA file format as the reference sequence input. In the beginning, the pipeline inspects the format of the input file. It is crucial to notice that in some intermediate steps of the pipeline (*e.g.,* in Mfold), long sequence titles might cause complications and should be avoided. In the homology search stage, BLASTn search is performed for each sequence in the selected subset of mature miRNAs as queries, against the input sequence(s) as the subject. Then, *LD* is computed for each aligned query-subject pair. In our pipeline, the default threshold for the maximum acceptable *LD* is set to 3 (although this parameter can be adjusted by the user).

**Extending the hit sequences and predicting their secondary structures**

After choosing a genomic region that has a certain level of similarity to some known miRNAs, a genomic window including this region together with *n* nucleotides on either side (*n*= 200 in the default settings) is selected using getFASTA. Then, this sequence is aligned against the comprehensive protein database. In AmiR-P^3^, it is possible to employ either BLASTx or DIAMOND for the alignment. We recommend using DIAMOND due to its faster algorithm, higher computing performance, and lower system resource requirements. To predict the RNA secondary structures of the sequences that were not similar to any protein dataset, we used one of the available prediction tools (namely Mfold [1], RNAfold [2], CONTRAfold [3], and MXFold2 [4]). Among these, Mfold is the recommended tool due to its efficient implementation algorithm and ability to predict multiple structures for a single RNA sequence.

**Feature extraction**

The CTAnalyzer software is basically developed to examine whether an RNA secondary structure has the typical characteristics of putative pri-miRNAs (Table 4). For each predicted structure, CTAnalyzer extracts 170 features (categorized in Table 5, and explained in Supplementary file S2). These structural features are employed by AmiR-P^3^ to distinguish real pri-miRNAs from other miRNA-resembling structures. CTAnalyzer also identifies the pre-miRNA sequences and determines their precise position on each of the predicted RNA structures. Since the length of flanking regions is preset, usually to a large value, the resulting sequences are often excessively lengthy, which may lead to an uninformative formation of multiple branches in the predicted secondary structures. That is to say, these multiple branches may not reflect the true structure, and consequently, the true function of the RNA molecule. To choose the pre-miRNA section, CTAnalyzer extracts a segment of the structure including the hit sequence, its complement, and the terminal stem-loop with two-nucleotide 3' overhangs.

**miRNA prediction**

A schematic illustration of a (predicted) secondary structure of an RNA sequence is demonstrated in Fig. S5.1. In the present work, we considered a secondary structure to be a potential plant pri-miRNA if the hit region and its complementary strand are both located in an uninterrupted stem with near-perfect complementarity (see Table 4), with no secondary branches or large internal loops in the suggested miR/miR* region, and a simple terminal loop (*i.e.,* no more than 3 terminal structures). CTAnalyzer can do an initial analysis to filter out the structures that do not have the basic properties of a primary miRNA. After this filtering procedure, all the remaining structures possess the general characteristics of a pri-miRNA. However, further investigation is required to distinguish genuine miRNA structures from miRNA-mimicking ones. Three further steps are then taken to achieve this goal: (1) Further inspecting the remaining structures based on more precise characteristics (such as the size, position, and distribution of mismatches, bulges, and internal loops in the secondary structure, the GC content and the free energy of the predicted pre-miRNA structure, *etc.*); (2) employing classification models, including the integrated deep learning classification model in AmiR-P^3^; and finally (3) a rule-based inspection of the qualified structures based on the miRNA sequence and structural characteristics (Table 4 in the main article).


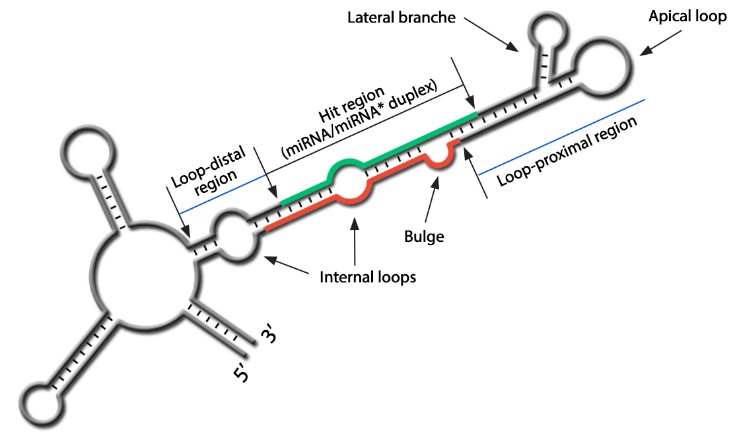


**Fig. S5.1.** Schematic illustration of a (predicted) secondary structure. As shown, the main branch of a predicted secondary structure is divided into three sections: the hit region (containing the hit sequence and its complement), the loop-proximal region (containing the apical loop, and the double-stranded stem which connects it to the hit region) and, the loop-proximal region (the un-branched double-stranded segment, downstream of the hit region). Each section may contain mismatches, internal loops, and bulges. In pre-miRNAs, lateral branches are also rarely seen, only in the loop-proximal region.

**References**

1. Zuker M. Mfold web server for nucleic acid folding and hybridization prediction. Nucleic Acids Research. 2003;31(13):3406-15.

2. Lorenz R, Bernhart SH, Höner zu Siederdissen C, Tafer H, Flamm C, Stadler PF, et al. ViennaRNA Package 2.0. Algorithms for Molecular Biology. 2011;6(1):26.

3. Do CB, Woods DA, Batzoglou S. CONTRAfold: RNA secondary structure prediction without physics-based models. Bioinformatics (Oxford, England). 2006;22(14):e90-e8.

4. Sato K, Akiyama M, Sakakibara Y. RNA secondary structure prediction using deep learning with thermodynamic integration. Nature communications. 2021;12(1):941.
