# Supplementary material for: AmiR-P3: An AI-based microRNA prediction pipeline in plants
Source: PLoS One. 2024 Aug 1;19(8):e0308016. doi: 10.1371/journal.pone.0308016 (PMC11293646; doi:10.1371/journal.pone.0308016)
Supplement: S7 File — The correlation between the features used in the training process presented as a heatmap. All of the supporting information is also available on GitHub at https://github.com/Ilia-Abolhasani/amir-p3/tree/master/supplementary%20information. (DOCX) [file pone.0308016.s010.docx]

**AmiR-P^3^: An AI-based microRNA prediction pipeline in plants**

Sobhan Ataei, Jafar Ahmadi, Sayed-Amir Marashi, Ilia Abolhasani

**S7 File**

**The correlation between the features used in the training process presented as a heatmap**

The heatmap analysis conducted in this study (Fig. S7.1) reveals insightful patterns within the features extracted from miRNA hairpin structures. The heatmap displays the Pearson correlation coefficients among three distinct feature groups: Structural features, Tetranucleotide features, and Connectivity features. Each group exhibits bi-clustered patterns, indicating cohesive relationships among the features within but minimal correlation between groups.

Structural features encompass parameters derived from the secondary structure of miRNA hairpins, such as mismatch size, loop size, and bulge characteristics, extracted using the CT-analyzer algorithm. Tetranucleotide features encode the composition of nucleotide sequences within the hit region, providing insights into sequence motifs. Connectivity features elucidate the interactions between nucleotides surrounding the hit region, shedding light on critical binding sites.

Notably, the heatmap demonstrates that while certain structural features exhibit high correlation, their inclusion remains valuable due to their ability to capture diverse aspects of the physical structure. This lack of detrimental correlation across feature groups suggests that the extracted features are informative and offer complementary perspectives without introducing redundancy. Consequently, these findings affirm the suitability of the feature set for training purposes, emphasizing the multifaceted nature of miRNA hairpin characteristics and their relevance in predictive modeling.

It should be noted that the decision to drop or retain correlated features is not straightforward and involves a trade-off between mitigating multicollinearity issues and potentially losing valuable information.

In our deep learning model for the classification task, we made careful choices regarding dropout rates, layer depths, and network architecture to effectively manage the impact of multicollinearity stemming from highly correlated features. By ensuring a well-structured network, we were able to achieve commendable results, even when dealing with a dataset that contained such correlated features. Deep learning models can also learn complex nonlinear relationships and interactions between features, and they use regularization techniques, such as dropout or batch normalization, which also reduce overfitting.

However, the question of dropping these correlated features is nuanced. On one hand, removing them can help alleviate multicollinearity-related problems that may hinder the convergence of the model during training. By doing so, we reduce the risk of the model's performance being influenced by redundant information. On the other hand, it is crucial to acknowledge that correlated features might collectively contribute to the underlying patterns of the data. These correlations might encapsulate meaningful relationships that are crucial for accurate predictions.

The decision to drop correlated features involves a careful balance between minimizing multicollinearity effects and retaining relevant information. To thoroughly investigate the impact of dropping correlated features, it would be ideal to conduct a future experiment, comparing the model's performance with and without these features. However, given the constraints of time and resources, such an investigation is not feasible within the scope of this current work.

In conclusion, while we have successfully managed to optimize our deep learning model for the given classification task by addressing multicollinearity through architectural choices, the question of dropping correlated features is a complex one. The decision ultimately hinges on the delicate balance between reducing multicollinearity-related issues and retaining essential information for accurate predictions. Future studies could explore the effects of this preprocessing step.

A


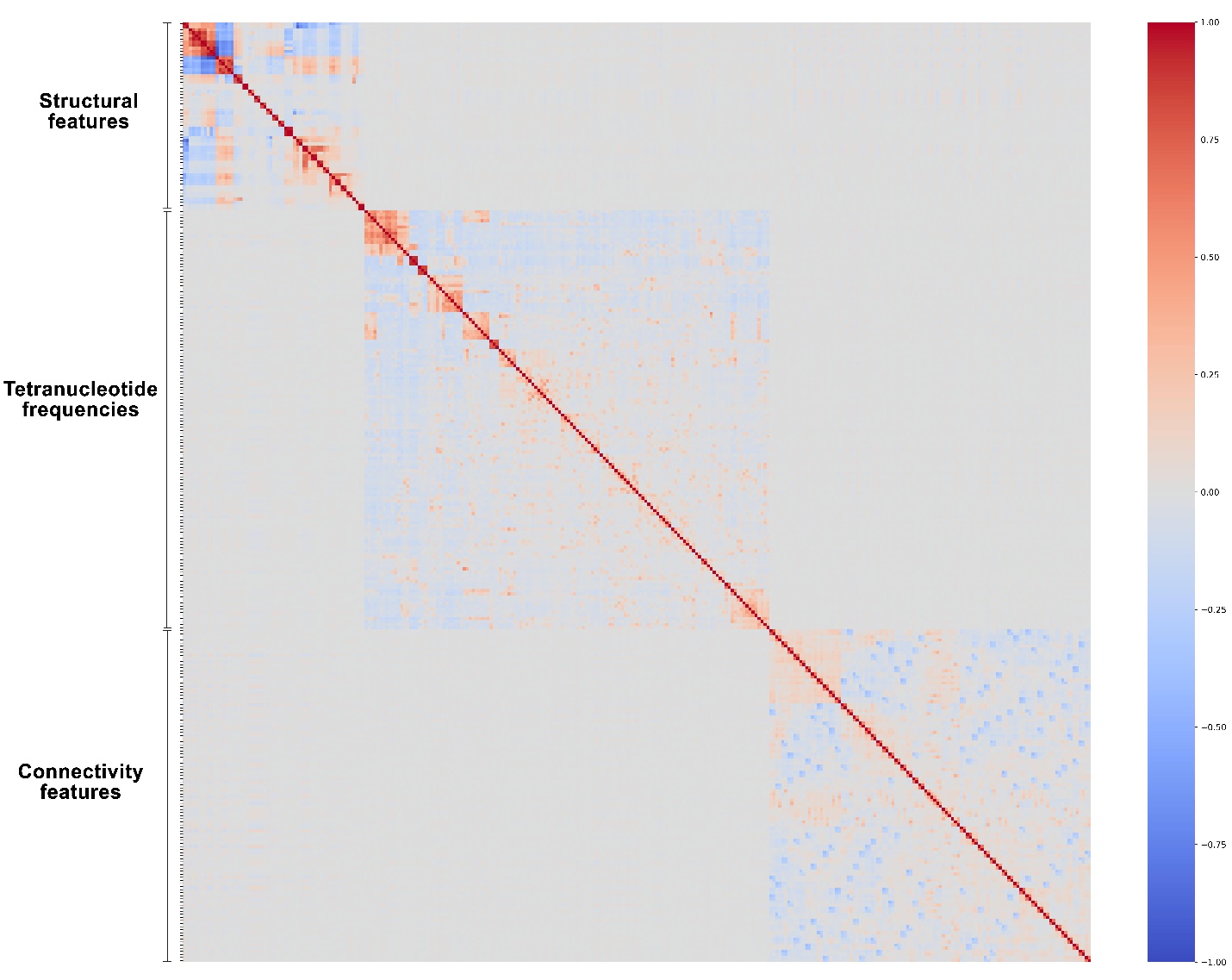


**
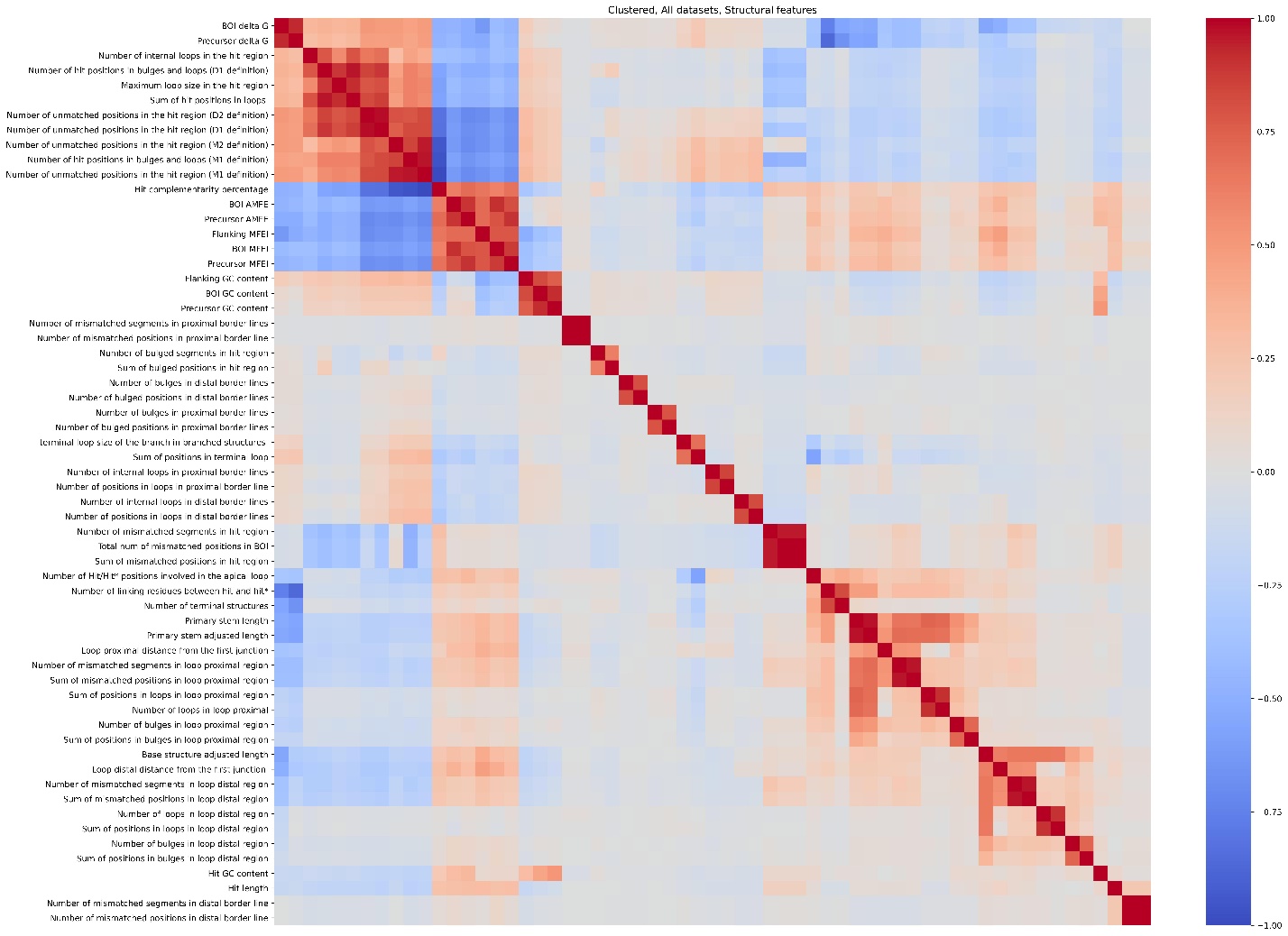
**

B

**
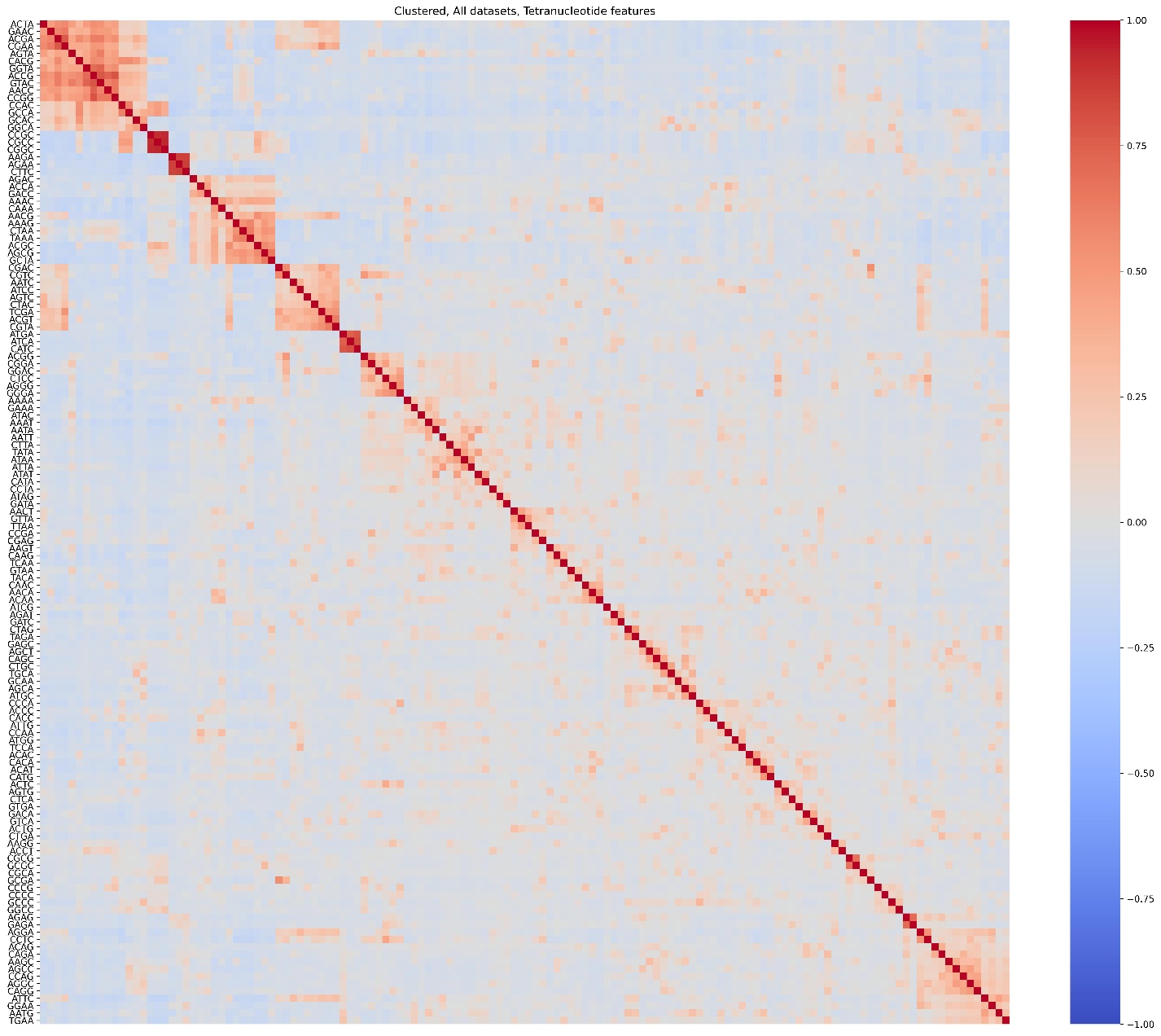
**

C

**
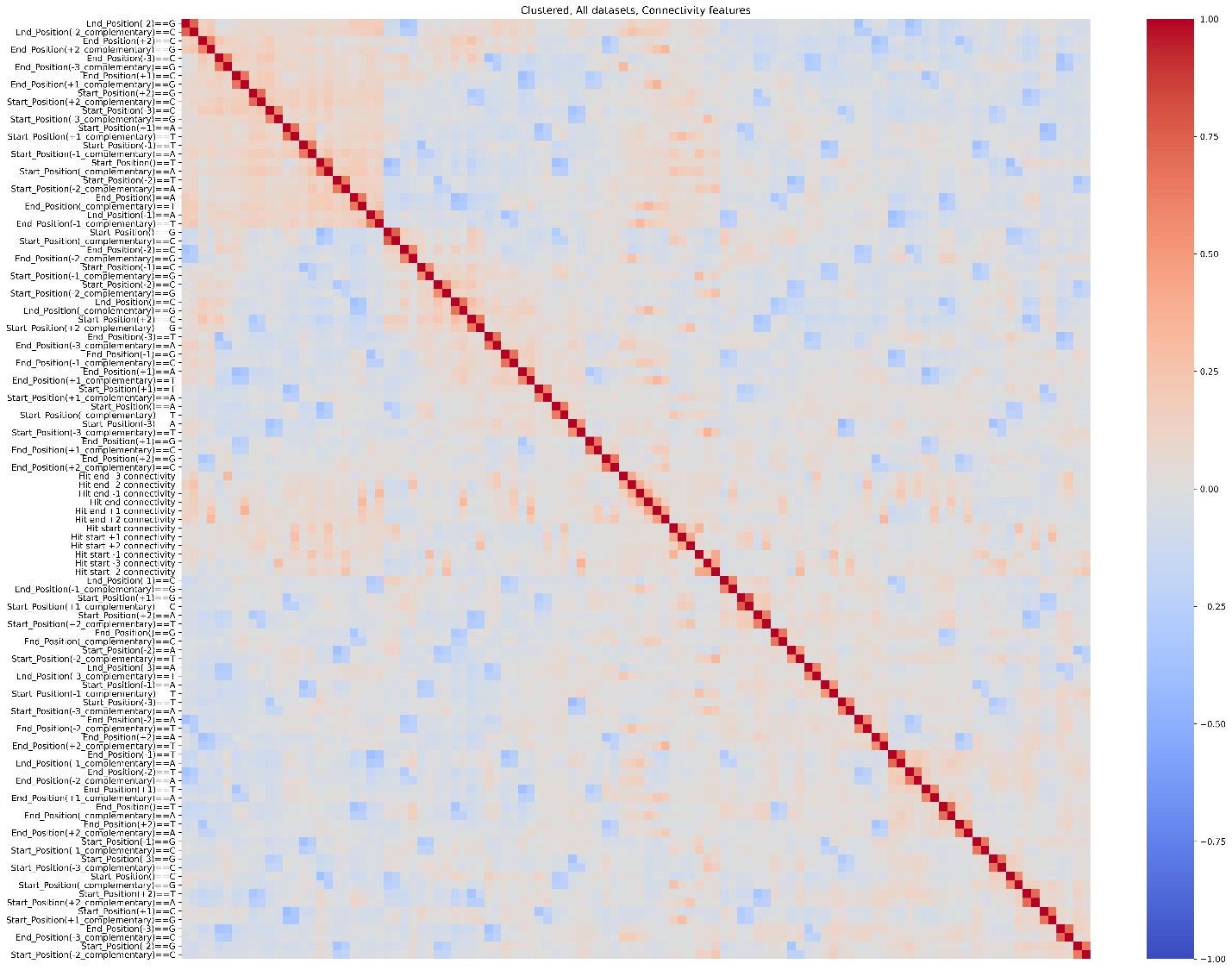
**

D

**Fig. S7.1.** Heatmap displaying Pearson correlation coefficients (A) among all of the features including (B) 61 structural features, (C) tetranucleotide frequencies (a 136-dimentional vector) and (D), 108 connectivity features extracted from miRNA hairpin structures, revealing cohesive relationships within feature groups and minimal correlation between groups.
