# Supplementary material for: AmiR-P3: An AI-based microRNA prediction pipeline in plants
Source: PLoS One. 2024 Aug 1;19(8):e0308016. doi: 10.1371/journal.pone.0308016 (PMC11293646; doi:10.1371/journal.pone.0308016)
Supplement: Attachment — Submitted filename: Comments @Ataei et al-2023-PLOSONE.docx [file pone.0308016.s011.docx]

The manuscript entitled “AmiR-P^3^: An AI-based microRNA prediction pipeline in plants” by Ataei *et al.* have developed a machine learning-based pipeline for plant miRNA prediction tool named AmiR-P^3^. The AmiR-P^3^ is an *ab initio* miRNA prediction tool with a requirement of minimalistic input, and also helpful in predicting miRNAs, even in less-studied plants. The authors identified many novel miRNAs in *Azadirachta indica*. The work is interesting and relevant, because it will help to predict miRNAs in newly sequenced plant genomes, those having no reference genome. However, the manuscript is well written but somewhere it does not very clear about the usage of parameters/tools. Following are some concerns which need to be addressed:

1. Authors stated that, to train a classifier to identify miRNAs, 2 datasets were used, one of known miRNAs and the other of miRNA-resembling sequences that are not miRNAs. For known miRNAs authors used miRBase, but did not explain from where they collected the transcripts resembled with pri-miRNA structures having overlaps with known coding sequences. Specify and explain about the negative dataset.
2. Authors must have to explain about the significance of taking LD value between 5 and 6.
3. The authors used DIAMOND software for alignment, though they are using BLAST, explain the usage priority of DIAMOND software.
4. Whether authors checked for tRNA or rRNA folds before qualifying non-coding sequences as pre-miRNAs predicted structures for positive dataset?
5. The authors mentioned that they have allowed six mismatched positions in the miRNA/miRNA* duplex, and mainly allowed three at asymmetric bulges. But it is assumed that the mismatches at position 9-11 will lead to non-functional miRNA. Whether authors checked the mismatch positions properly?
6. Why authors compared AmiR-P3 only with PlantMirP2? Why not with other miRNA prediction tools?
