# Supplementary material for: AmiR-P3: An AI-based microRNA prediction pipeline in plants
Source: PLoS One. 2024 Aug 1;19(8):e0308016. doi: 10.1371/journal.pone.0308016 (PMC11293646; doi:10.1371/journal.pone.0308016)
Supplement: Attachment — Submitted filename: Response to Reviewers.docx [file pone.0308016.s012.docx]

**Dear Editor,**

Thank you for your feedback on our manuscript. We have thoroughly reviewed the reference list as per your suggestion and can confirm that none of the cited papers have been retracted. Therefore, there are no retracted articles in the reference list that require specific attention or action.

Best regards,

Sobhan Ataei

**Dear Reviewers,**

Thank you for dedicating your valuable time and expertise to evaluate my manuscript entitled "AmiR-P^3^: An AI-based microRNA prediction pipeline in plants" for publication in PLoS ONE. I am grateful for your insightful feedback and suggestions, which have greatly contributed to enhancing the quality of the manuscript.

I have carefully considered each of your comments and have diligently incorporated the requested revisions into the manuscript. These modifications have been clearly marked with tracking in the Word document accompanying this letter. Please review these changes at your convenience and do not hesitate to reach out if you require further clarification.

I am confident that the revised manuscript now meets the high standards expected for publication. Your constructive input has been invaluable in this process, and I am eager to receive your final assessment. Thank you once again for your assistance, and I eagerly anticipate your response.

Best regardes,

Sobhan Ataei

**Reviewer #2**

**Comment #1:** Authors should at least present the correlation between features as figure or table or heatmap to guide.

Answer: Thank you for your valuable feedback and suggestions on our manuscript “AmiR-P3: An AI-based microRNA prediction pipeline in plants". We appreciate your thorough review and the opportunity to improve our work. Regarding your suggestion to present the correlation between features used in the training process, we have carefully considered your comment and have incorporated a heatmap illustrating these correlations. This heatmap can be found in Supplementary File S7, which we have newly included with the revised submission. We would like to note that Supplementary File S7 is referenced in both line 608 and line 803 of the revised manuscript. We believe that this addition provides a clear and informative visualization to guide readers through the relationships between the features analyzed in our study.

**The added text (lines 608 to 609 of the revised manuscript):**

The correlation between the features used in the training process is presented in S7 File as a heatmap.

**Comment #2:** Overview of available tool should come in introduction and the lacuna the authors are addressing should follow intermediately after that.

Answer: Thank you for your valuable suggestion to provide an overview of available tools related to the topic at the beginning of the introduction. We have now included this overview (lines 85 to 100 in the revised manuscript) to better contextualize the research and highlight the existing tools in the field. Limits of currently available software tools are discussed in lines 101 to 140, and advantages of the introduced miRNA prediction tool are briefly addressed in lines 141 to 148. Your feedback has been instrumental in improving the clarity and structure of the manuscript.

**The added text (lines 85 to 102 of the revised manuscript):**

In the field of plant microRNA prediction, several computational tools have been developed to aid researchers in identifying and characterizing these small regulatory molecules. Tools such as miRNAFinder [32], PlantMirP2 [33], mirMachine [34], PmiRDiscVali [35], miRDeep-P2 [36], SUmir [37], and miRNA Digger [38] have been instrumental in advancing our understanding of plant microRNAs. miRNAFinder utilizes machine learning algorithms to predict novel microRNAs from small RNA sequencing data. PlantMirP2 is a comprehensive tool that integrates multiple prediction algorithms to identify plant microRNAs and their targets. mirMachine employs a homology-based approach for accurate microRNA prediction. PmiRDiscVali focuses on validating predicted plant microRNAs through experimental data analysis. miRDeep-P2 is a widely used tool for discovering known and novel microRNAs from deep sequencing data. SUmir offers a user-friendly interface for predicting microRNAs and their targets in plants. Finally, miRNA Digger is a versatile tool that allows for the identification of conserved and species-specific microRNAs in plant genomes. These tools play a crucial role in expanding our knowledge of plant microRNAs and their regulatory functions, providing valuable insights into the intricate networks governing plant development and stress responses.

Although there are numerous plant miRNA prediction tools available, the majority of them exhibit limitations in terms of flexibility across three critical dimensions:

**Comment #3:** Author must make there training data available for reproducible research.
I am unable to access <https://hub.docker.com/r/micrornaproject/amir-p3>, authors must verify that the link is working.

Answer: We appreciate your interest in ensuring the transparency and replicability of our work. We would like to address your concern by confirming that all the necessary data, including the training datasets, required to reproduce the research findings are readily available on our GitHub repository at "https://github.com/Ilia-Abolhasani/amir-p3". Additionally, the software developed during the course of this study, named "AmiR-P^3^" has been made accessible to researchers through the Docker platform at "https://hub.docker.com/r/micrornaproject/amir-p3". We believe that providing access to both the training data and the developed plant microRNA prediction software tool developed in our research will facilitate the replication and validation of our results by the scientific community. The link to the developed software tool and the training datasets are now available in the revised manuscript (line 44 and line 46 respectively).

**The corrected text #1 (lines 41 to 46 of the revised manuscript):**

AmiR-P3 is provided as a docker container, which is a portable and self-contained software package that can be readily installed and run on any platform, and is freely available for non-commercial use from:

https://hub.docker.com/r/micrornaproject/amir-p3

The source code of AmiR-P3 is also freely available from:

https://github.com/Ilia-Abolhasani/amir-p3

**The corrected text #2 (lines 356 to 361 of the revised manuscript):**

For simplicity, we provide AmiR-P^3^ as a Docker container (freely available from <https://hub.docker.com/r/micrornaproject/amir-p3>), allowing the users to install the pipeline in a single step without the need to pre-install any other utilities. The source code of AmiR-P^3^ and all the other data neccesarry to make the research reproducible (including the training datasets) are freely available from <https://github.com/Ilia-Abolhasani/amir-p3> .

**Comment #4:** English and grammar should be checked thoroughly.

Answer: We appreciate your comments and have carefully reviewed the manuscript to address the English and grammar issues. We have made revisions in line 37, 59, 83, 116, 118, 121, 131, 177, 188, 195, 212, 232, 235, 275, 307, 311, 350, 358, 432, 462, 516, 522, 530, 550, and 553 (in the revised manuscript) to ensure the manuscript meets high linguistic standards. The track changes version of the article reflects these corrections, ensuring clarity and correctness throughout the text. We hope that the revised manuscript now meets your expectations regarding language quality.
